# Supplementary material for: Residual-aware health prediction of power transformers via spatiotemporal graph neural networks
Source: PLoS One. 2025 Nov 10;20(11):e0332381. doi: 10.1371/journal.pone.0332381 (PMC12599914; doi:10.1371/journal.pone.0332381)
Supplement: S1 Text — (PDF) [file pone.0332381.s001.pdf]

%The main procedure of this article is as follows. Please contact the correspondent if it is complete.

```
clear; clc; close all;

%% 1. Parameters and Setup
T = 1440;                  % Number of time steps (e.g., 1 day at 1-min interval)
n = 18;                    % Number of sensors (nodes)
fs = 1;                    % Sampling frequency (1/min)
time = (1:T)' / fs;

% Sensor groupings (representing transformer subsystems)
subsystems = {
        'Winding',        1:4;
        'Core',           5:8;
        'Cooling',        9:12;
        'Insulation',    13:15;
        'TapChanger',    16:18;
};
```

```
%% 2. Generate SCADA-like multivariate sensor
signals
X = zeros(T, n);    % SCADA data matrix: [T x n]

for i = 1:n
    base = 60 + 5 * sin(2*pi*time/1440);                %
diurnal baseline
    noise = 0.5 * randn(T, 1);                          %
sensor noise
    fault = zeros(T,1);

    if mod(i, 6) == 0
        % Inject fault into some variables: slow drift
from t=1000 to 1100
        fault(1000:1100) = linspace(0, 20, 101)';
    end

    X(:,i) = base + noise + fault;
end

%% 3. Plot a few raw signals
figure;
```

```
plot(time, X(:,[1 6 13]), 'LineWidth', 1.2);
legend('Winding T1','Cooling Sensor','Insulation Gas');
title('Simulated Transformer Sensor Signals');
xlabel('Time (min)'); ylabel('Value');
grid on;

%% 4. Compute residuals from moving average forecast (unsupervised model)
L = 12;    % Window length
X_hat = movmean(X, [L-1 0]);    % Moving average as naive prediction
residuals = abs(X - X_hat);    % Pointwise residuals

%% 5. Compute Health Indicator (HI) and detect anomalies
HI = mean(residuals, 2);    % Mean residual over all sensors
threshold = quantile(HI, 0.95);    % 95% quantile threshold
anomaly_index = find(HI > threshold);    %
```

Detected fault index

```
%% 6. Plot the Health Indicator
figure;
plot(HI, 'b'); hold on;
yline(threshold, 'r--', 'Threshold (95%)');
plot(anomaly_index, HI(anomaly_index), 'ro');
title('Health Indicator (HI) and Fault Detection');
xlabel('Time Step'); ylabel('HI Value');
legend('Health Indicator','Threshold','Detected Fault');
grid on;

%% 7. Summary
fprintf('Total anomalies detected: %d\n', numel(anomaly_index));
fprintf('Threshold (95%% quantile): %.3f\n', threshold);
function A = graph_adjacency_generator(X)
% Generate correlation-based graph adjacency matrix
% Input:
%     X: [T x n] time-series data of n variables
% Output:
```

```
%      A: [n x n] adjacency matrix (Pearson correlation)

n = size(X, 2);
A = corrcoef(X);
A(isnan(A)) = 0;        % Replace NaN due to zero
variance
A = abs(A);                  % Use absolute correlation as
edge strength

% Optional: apply threshold
threshold = 0.3;
A(A < threshold) = 0;

End
function H = chebyshev_gcn_layer(X, A, K, W)
% Chebyshev Graph Convolution Layer (K-order)
% Inputs:
%      X: [n x d] node features
%      A: [n x n] adjacency matrix
%      K: order of Chebyshev polynomial
%      W: [d x out_dim x K] learnable weights
% Output:
```

```
%      H: [n x out_dim] output features

n = size(X, 1);
d = size(X, 2);
out_dim = size(W, 2);

% Normalize Laplacian
D = diag(sum(A, 2));
L = D - A;
L_tilde = 2 * L / max(eig(L)) - eye(n);    % scaled

T_k = cell(K, 1);
T_k{1} = X;
if K > 1
        T_k{2} = L_tilde * X;
        for k = 3:K
                T_k{k} = 2 * L_tilde * T_k{k-1} - T_k{k-2};
        end
end

% Compute output
```

```
    H = zeros(n, out_dim);
    for k = 1:K
        for j = 1:out_dim
            H(:, j) = H(:, j) + T_k{k} * W(:, j, k);
        end
    end
    end
function plot_HI_with_anomaly(HI, threshold, anomaly_idx, time)
    % Plot HI curve and highlight detected anomalies
    % Inputs:
    %    HI: [T x 1] health indicator
    %    threshold: scalar
    %    anomaly_idx: indices where HI exceeds threshold
    %    time: optional time vector

    if nargin < 4
        time = 1:length(HI);
    end

    figure;
```

```
plot(time, HI, 'b', 'LineWidth', 1.2); hold on;
yline(threshold, 'r--', 'Threshold');
plot(time(anomaly_idx), HI(anomaly_idx), 'ro');
xlabel('Time'); ylabel('HI Value');
legend('Health Indicator','Threshold','Detected Fault');
title('Residual-Based Health Indicator with Anomaly Detection');
grid on;

end
```
