# Supplementary material for: Residual-aware health prediction of power transformers via spatiotemporal graph neural networks
Source: PLoS One. 2025 Nov 10;20(11):e0332381. doi: 10.1371/journal.pone.0332381 (PMC12599914; doi:10.1371/journal.pone.0332381)
Supplement: Attachment — Submitted filename: Response to Reviewers.pdf [file pone.0332381.s003.pdf]

Response to Reviewer No. 1:

Ms. No. PONE-D-25-34707

"Residual-Aware Health Prediction of Power Transformers via Spatiotemporal Graph Neural Networks"

We greatly appreciate the reviewers' efforts in evaluating our manuscript. Based on the feedback received, we have revised our document to improve both the accuracy and clarity. To assist the reviewers in identifying these changes, we have highlighted the sections revised in response to **Reviewer 1's comments** in **red**. Thank you for the constructive insights that have helped enhance our work.

1. **Comment 1:** *"The statement of the problem is not adequate, and the manuscript does not clearly describe how it is similar to or different from existing research. In particular, there are numerous related studies on fault detection of power transformers combined with graph neural networks, and the paper should position itself clearly within this context."*

   Response: We thank the reviewer for highlighting that the problem statement in the original manuscript did not clearly articulate the similarities and differences between our work and existing research. In response, we have made three substantive improvements in the Introduction: (1) We have explicitly defined the research focus and core problem, framing "transformer health prediction and anomaly detection" as the task of jointly modeling *spatial dependencies* (across components/sensors) and *temporal dependencies* in multivariate SCADA sequences under nonstationary operating conditions, with a *residual-driven health indicator* enabling early and interpretable alarms;

   (2) We have clarified the positioning of our approach relative to over one hundred related studies in the field (including GCN-, GAT-, and dynamic-graph-based models, as well as sequence-only anomaly detection methods), adding direct comparative statements with representative methods in three categories: static-graph GCN-type, dynamic/attention-based, and purely temporal models. We highlight three common limitations in prior work: static or heuristic adjacency definitions, insufficient residual-based diagnostics/interpretability, and weak adaptability to nonstationary distribution shifts;

   (3) We have rewritten the contributions as *comparable and verifiable* technical points, each corresponding to a specific design element—dynamic graph learning, Chebyshev spectral graph convolution, residual-enhanced GRU, and quantile-thresholded HI—and contrasted them *component-by-component* with the most relevant prior works (e.g., static GCN, GAT/dynamic-graph, StemGNN, TempGNN).

   These changes have been incorporated into the revised Introduction, with all added or modified content marked in red in the manuscript to facilitate review.

   Modification:

   Specifically, this study considers the practical setting where transformer health must be *forecast* and *assessed* under nonstationary operating regimes by jointly modeling (i) spatial couplings among subsystem-related sensor variables and (ii) temporal dynamics in

multivariate SCADA sequences, while offering residual-driven indicators for early and interpretable fault alarms.

Therefore, sequence-only models are prone to overlook cross-component interactions (e.g., thermal–electromagnetic couplings among oil, windings, and core), which are crucial for robust state prediction and timely anomaly localization in power transformers.

In particular, (a) static or heuristic adjacency often underfits evolving dependencies in nonstationary SCADA streams; (b) dynamic/attention graphs improve flexibility but are seldom coupled with residual-aware temporal decoders tailored for health assessment; and (c) many approaches emphasize point prediction accuracy while providing limited, model-consistent indicators for interpretable early warning. These gaps, identified through a comprehensive survey of the transformer fault detection literature leveraging GNNs and related spatiotemporal models (over one hundred studies accessible via major scholarly databases), motivate the integrated design presented in this work.

Compared with *static-graph* GCN-based monitors [9, 10], our approach *learns* time-varying dependencies online; relative to *attention/dynamic* graph learners [11, 12], we explicitly fuse Chebyshev spectral filtering with a residual-enhanced GRU decoder to stabilize and sharpen temporal error signals; and unlike sequence-only baselines (e.g., LSTM/TCN/Transformer) [4, 5, 6], we provide a residual-based HI with quantile thresholds that aligns prediction uncertainty with actionable anomaly alarms.

Paper organization. Section 2 introduces the transformer subsystems and the observability-driven state design. Section 3 details the dynamic graph construction, Chebyshev spectral graph convolution, and residual-enhanced temporal modeling, and defines the residual-based HI. Section 4 presents datasets, baselines, training protocols, and statistical analyses. Section 5 reports forecasting and detection results with ablations and robustness tests, followed by conclusions in Section 6.

2. **Comment 2:** *"In the section on the Design of Spatiotemporal Graph Neural Network for Health Status Prediction, the description lacks adequate reference to existing literature and does not sufficiently justify the proposed approach. As a result, the claims are not convincingly supported."*

   Response: We thank the reviewer for pointing out this important issue. In the original manuscript, Section 3 did not sufficiently link our model design choices to prior studies, nor did it provide enough justification for each architectural component. To address this, we have substantially revised the section by:

   (1) Adding targeted references to representative works in each relevant domain—dynamic graph construction [11, 12, 18], Chebyshev spectral graph convolution [13, 14], and residual-enhanced GRU temporal modeling [15, 16];

   (2) Explicitly explaining why each component is selected for transformer health state forecasting under nonstationary SCADA data conditions, supported by empirical evidence from the literature;

   (3) Providing a comparative discussion highlighting how our integrated design addresses the limitations of static-graph GCNs, pure temporal models, and dynamic-graph approaches without residual-based temporal decoding.

These changes strengthen the theoretical foundation of our approach and make the design rationale more transparent. All new references and explanatory sentences are marked in red in the revised manuscript.

Modification: In Section 3.1, we added:

Our design choices are motivated by prior evidence in related domains: attention-based dynamic graph construction has been shown to improve adaptability to evolving dependencies in nonstationary sensor networks [11, 12, 18]; Chebyshev spectral graph convolution [13, 14] provides extended receptive fields and computational efficiency for graph-structured industrial data; and residual-enhanced GRUs [15, 16] improve temporal stability and sensitivity to subtle system drifts. By integrating these components, our STGNN addresses three common limitations in existing works: (1) static-graph models cannot adapt to changing dependencies, (2) sequence-only models neglect cross-variable structure, and (3) dynamic-graph methods without residual-aware decoders lack interpretable, error-driven health indicators.

The overall framework consists of three main components:

In our implementation, the GNN layer is instantiated as a Chebyshev spectral graph convolution [13, 14], enabling efficient localized spectral filtering with a polynomial order of $K = 3$. This choice allows the model to capture higher-order neighborhood information without incurring the high computational cost of full spectral decomposition, which is particularly advantageous for medium-scale transformer monitoring graphs.

The sequence of graph embeddings is then passed through a GRU network to learn temporal dependencies:

$$\mathbf{h}_t^{\text{pred}} = \text{GRU}(\mathbf{H}_{t-L+1}, ..., \mathbf{H}_t),$$

The GRU module is augmented with residual connections [15, 16], which stabilize gradient flow and improve the model's sensitivity to subtle yet persistent deviations—often early indicators of equipment degradation. The resulting temporal representation is subsequently fed into a fully connected layer to produce the predicted state estimate for the next time step:

$$\hat{\mathbf{X}}_{t+1} = \text{FC}(\mathbf{h}_t^{\text{pred}}).$$

Based on the predicted and observed states, a root mean square error (RMSE)-based health indicator is computed and compared against an empirical threshold to judge whether an anomaly has occurred. We further derive a residual-based health indicator (HI) by aggregating node-wise squared errors, and employ quantile-based thresholding [19, 20] to trigger early and interpretable alarms. This direct linkage between prediction residuals and anomaly alerts distinguishes our approach from many existing GNN-based forecasting models, which often lack a principled mechanism for fault detection.

3. **Comment 3:** *"The implementation details are not adequately described, making it difficult for readers to fully understand, evaluate, or replicate the work."*

   Response: We thank the reviewer for this valuable comment. In the original manuscript, we recognize that the implementation details provided were insufficient for full comprehension and reproducibility. To address this, we have expanded the experimental setup

section and added an explicit "Implementation Details" subsection that specifies: (1) software environment (frameworks, versions, and hardware specifications); (2) hyperparameter configurations, including learning rate, optimizer settings, batch size, number of epochs, and early stopping criteria; (3) graph construction parameterization, including polynomial order K for Chebyshev convolution and weight coefficients $(\lambda_1, \lambda_2, \lambda_3)$ for combining static, Gaussian, and attention-based adjacency matrices; (4) data preprocessing procedures, including normalization, missing data handling, and sliding window segmentation; and (5) model training pipeline, including random seed setting for reproducibility and evaluation metrics computation.

These additions ensure that the method can be independently implemented and verified by other researchers. All newly added details are marked in red in the revised manuscript.

Modification: In Section 4.1 "Experimental Setup and Data Description," we have added:

*"**Implementation Details.** All experiments were implemented in Python 3.10 using PyTorch 2.1 and the Deep Graph Library (DGL) 1.1, and conducted on a workstation with an NVIDIA RTX 3090 GPU (24GB), AMD Ryzen 9 5950X CPU, and 64GB RAM. Model training employed the Adam optimizer with an initial learning rate of 0.001, batch size of 64, and early stopping patience of 20 epochs based on validation loss. The Chebyshev polynomial order was set to $K = 3$ for spectral graph convolution, and the adjacency matrix was formed by a weighted combination of static Pearson correlation, Gaussian kernel similarity, and attention-based dynamic edges with $\lambda_1 = 0.3$, $\lambda_2 = 0.3$, and $\lambda_3 = 0.4$. Input data were normalized via Z-score transformation, missing values were linearly interpolated, and a sliding window of length $L = 12$ was applied to form model inputs. Random seeds were fixed across all runs to ensure reproducibility. Evaluation metrics, including RMSE, MAPE, and Max-Error for forecasting, and Accuracy, Precision, Recall, and F1-score for anomaly detection, were computed following standard definitions."*

4. **Comment 4:** *"The claims of contributions are too general and asserted without sufficient comparative evidence. The paper should compare with the most relevant studies and make specific, evidence-based claims."*

   Response: We thank the reviewer for highlighting this important issue. In the original version, the contributions section presented abstract claims without sufficiently grounding them in experimental comparisons. To address this, we have revised the contribution statements in the Introduction to be (1) more specific in describing each methodological innovation, and (2) directly supported by evidence from our benchmarking experiments against four representative baseline models (LSTM, GCN-LSTM, StemGNN, and TempGNN), as shown in Table 2 and Figure 2–3.

   Each contribution is now aligned with the corresponding architectural component (e.g., dynamic attention-based graph construction, Chebyshev convolution, residual-enhanced GRU, and quantile-thresholded health indicator), and we quantify performance gains in prediction and detection tasks using RMSE, MAPE, and F1-score metrics.

   These revisions clarify both the novelty and practical effectiveness of our design. The revised contribution bullets are marked in red in the updated Introduction.

   Modification: In the Introduction, we have revised the contributions section as follows:

- Transformer health monitoring is formulated as a dynamic spatiotemporal graph learning problem, where sensor dependencies are captured at each time step using attention-based mechanisms. The limitations of static graph models in adapting to varying operational conditions are addressed.
- A hybrid architecture is proposed by integrating Chebyshev spectral graph convolution with residual-enhanced GRUs, allowing long-range spatial dependencies and stable temporal patterns to be effectively modeled.
- A residual-based health indicator is defined and a quantile-based thresholding strategy is employed, enabling early anomalies to be detected in an interpretable and real-time manner.
- A synthetic SCADA benchmark covering multiple transformer subsystems and fault scenarios is constructed, and the proposed method is evaluated through comparative experiments against representative baseline models.

References

[1] Zhao R, Yan R, Chen Z, Mao K, Wang P, Gao R. Deep learning and its applications to machine health monitoring. *Mechanical Systems and Signal Processing*. 2019;115:213–237.

[2] Li Y, Liu S, Shu L. Wind turbine fault diagnosis based on Gaussian process classifiers applied to operational data. *Renewable Energy*. 2019;134:357–366.

[3] Li C, Liu D, Wang M, Wang H, Xu S. Detection of outliers in time series power data based on prediction errors. *Energies*. 2023;16(2):582.

[4] Kim D-K, Kim K. A convolutional transformer model for multivariate time series prediction. *IEEE Access*. 2022;10:101319–101329.

[5] Cui Z, Ke R, Pu Z, Wang Y. Deep bidirectional and unidirectional LSTM networks for network-wide traffic speed prediction. arXiv preprint arXiv:1801.02143. 2018.

[6] Li Y, Yu R, Shahabi C, Liu Y. Diffusion convolutional recurrent neural network: Data-driven traffic forecasting. In: *Proc. ICLR*; 2018.

[7] Wu Z, Pan S, Chen F, Long G, Zhang C, Philip SY. A comprehensive survey on graph neural networks. *IEEE Trans Neural Netw Learn Syst*. 2021;32(1):4–24.

[8] Zhou J, Cui G, Zhang Z, Yang C, Liu Z, Wang L, Li C, Sun M. Graph neural networks: A review of methods and applications. *AI Open*. 2020;1:57–81.

[9] Li Y, Xue C, Zargari F, Li YR. From graph theory to graph neural networks (GNNs): The opportunities of GNNs in power electronics. *IEEE Access*. 2023;11:145067–145084.

[10] Cao Y, Tang X, Deng X, Wang P. Fault detection of complicated processes based on an enhanced transformer network with graph attention mechanism. *Process Safety and Environmental Protection*. 2024;186:783–797.

[11] Wang X, Han H, Gao B. Enhancing power transformer fault diagnosis through dynamic knowledge graph reasoning. *IEEE Trans Instrum Meas*. 2024;[Online early access].

[12] Cai L, Chen Z, Luo C, Gui J, Ni J, Li D, Chen H. Structural temporal graph neural networks for anomaly detection in dynamic graphs. In: *Proc. 30th ACM Int Conf on Information & Knowledge Management (CIKM)*; 2021. p. 3747–3756.

[13] Kipf TN, Welling M. Semi-supervised classification with graph convolutional networks. In: *Proc. ICLR*; 2017.

[14] Yu B, Yin H, Zhu Z. Spatio-temporal graph convolutional networks: A deep learning framework for traffic forecasting. In: *Proc. IJCAI*; 2018. p. 3634–3640.

[15] Bloemheuvel S, van den Hoogen J, Jozinović D, Michelini A, Atzmueller M. Graph neural networks for multivariate time series regression with application to seismic data. *Int J Data Sci Anal.* 2023;16(3):317–332.

[16] Rolander A, Ter Vehn A, Eriksson R, Nordström L. Real-time transient stability early warning system using graph attention networks. *Electric Power Systems Research.* 2024;235:110786.

[17] Chanda D, Soltani NY. A heterogeneous graph-based multi-task learning for fault event diagnosis in smart grid. *IEEE Trans Power Syst.* 2024;[Online early access].

[18] Li ZL, Zhang GW, Yu J, Xu LY. Dynamic graph structure learning for multivariate time series forecasting. *Pattern Recognition.* 2023;138:109423.

[19] Maldonado-Correa J, Torres-Cabrera J, Martín-Martínez S, Artigao E, Gómez-Lázaro E. Wind turbine fault detection based on the transformer model using SCADA data. *Engineering Failure Analysis.* 2024;162:108354.

[20] Tariq S, Tariq S, Kim S, Woo SS, Yoo C. Distance adaptive graph convolutional gated network-based smart air quality monitoring and health risk prediction in sensor-devoid urban areas. *Sustainable Cities and Society.* 2023;91:104445.

[21] Fassi Y, Heiries V, Boutet J, Boisseau S. Toward physics-informed machine-learning-based predictive maintenance for power converters—a review. *IEEE Trans Power Electron.* 2023;39(2):2692–2720.

[22] Tian J, Shen C, Wang B, Ren C, Xia X, Dong R, Cheng T. EVADE: Targeted adversarial false data injection attacks for state estimation in smart grid. *IEEE Trans Sustain Comput.* 2024;[Online early access].

[23] VS A, et al. Multi-label deep learning classification approach for false data injection attacks in smart grid. *KSII Trans Internet Inf Syst.* 2021;15(6).

[24] Tian J, Wang B, Wang Z, Cao K, Li J, Ozay M. Joint adversarial example and false data injection attacks for state estimation in power systems. *IEEE Trans Cybern.* 2021;52(12):13699–13713.

The authors would like to express our sincere appreciation to the referee for his/her constructive yet critical comments and the efforts provided in helping us to improve the quality and presentation of the paper further.

Response to Reviewer No. 3:

Ms. No. PONE-D-25-34707

"Residual-Aware Health Prediction of Power Transformers via Spatiotemporal Graph Neural Networks"

We sincerely appreciate the time and effort the reviewers have invested in assessing our manuscript. In response to the feedback provided, we have made revisions to enhance both the precision and comprehensibility of our document. To facilitate the reviewers' assessment of these changes, we have marked the sections modified in response to **Reviewer 3's comments** in **red**. We are grateful for the valuable insights that have significantly improved our manuscript.

1. **Comment 1:** *"In Introduction, a paragraph should be added to introduce the structure of the paper."*

   Response: We thank the reviewer for the helpful suggestion. To improve the readability and logical flow of the paper, we have added a paragraph at the end of the Introduction that briefly outlines the structure of the manuscript. This paragraph serves to guide readers through the main sections and their respective content.

   Modification: At the end of the Introduction, the following paragraph has been added and marked in red:

   "The remainder of this paper is organized as follows. Section 2 introduces the transformer system structure and describes the construction of observability-driven health state vectors. Section 3 presents the proposed STGNN framework, including dynamic graph construction, spectral graph convolution, residual-enhanced temporal modeling, and the health indicator design. Section 4 details the experimental setup, data generation process, and baseline methods. Section 5 reports the results of forecasting and anomaly detection experiments, along with comparative analyses. Finally, Section 6 concludes the paper and outlines limitations and future research directions."

2. **Comment 2:** *"The formulas in the article are missing numbering."*

   Response: We thank the reviewer for pointing out this oversight. In the original manuscript, several key formulas appeared without numbering, which may have caused inconvenience for reference and discussion. In the revised manuscript, we have reviewed all mathematical expressions and added consistent equation numbers to every major formula. These include graph convolution operations, GRU update rules, prediction equations, health indicator definitions, and loss functions. This ensures that each formula can now be explicitly referenced throughout the text.

   Modification: All mathematical expressions in this paper now include equation numbers.

3. **Comment 3:** *"Since there are some papers in this topic, the contributions of the manuscript should be better summarized and listed."*

   Response: We thank the reviewer for this constructive suggestion. In the original submission, the contributions were described in a general manner and lacked structural clarity. To address this, the contributions in the Introduction have been rewritten into a concise

and clearly enumerated list. Each item reflects a specific methodological innovation or evaluation effort and is framed in passive voice. The revised contributions are also better aligned with related literature and highlight how our proposed framework differs from existing work in terms of modeling dynamics, robustness, and interpretability.

Modification: At the end of the Introduction, the contributions have been updated as follows (marked in red in the manuscript):

- Transformer health monitoring is formulated as a dynamic spatiotemporal graph learning problem, where sensor dependencies are captured at each time step using attention-based mechanisms. The limitations of static graph models in adapting to varying operational conditions are addressed.
- A hybrid architecture is proposed by integrating Chebyshev spectral graph convolution with residual-enhanced GRUs, allowing long-range spatial dependencies and stable temporal patterns to be effectively modeled.
- A residual-based health indicator is defined and a quantile-based thresholding strategy is employed, enabling early anomalies to be detected in an interpretable and real-time manner.
- A synthetic SCADA benchmark covering multiple transformer subsystems and fault scenarios is constructed, and the proposed method is evaluated through comparative experiments against representative baseline models.

4. **Comment 4:** *"While the introduction sets the context, a more explicit literature review section could better situate the study within the broader research landscape, such as Residual-based attention Physics-informed Neural Networks for spatio-temporal ageing assessment of transformers operated in renewable power plants, EVADE Targeted Adversarial False Data Injection Attacks for State Estimation in Smart Grid, LESSON Multi-Label Adversarial False Data Injection Attack for Deep Learning Locational Detection, Joint Adversarial Example and False Data Injection Attacks for State Estimation in Power Systems, and so on."*

   Response: We sincerely appreciate the reviewer's suggestion to provide a more explicit and comprehensive literature review. While our original Introduction provided some general background, we agree that a dedicated section better highlighting recent and thematically relevant work would improve the contextual framing of our contribution. Accordingly, a new subsection titled **"Related Work"** has been added at the end of Section 1. In this subsection, we have incorporated and discussed the representative references suggested by the reviewer, including studies on residual-based physics-informed networks, adversarial robustness in smart grids, and false data injection detection. We also explain how our work differs from and complements these prior studies by focusing on residual-driven health indicators under dynamic spatiotemporal graph learning, rather than purely adversarial or physics-informed estimation.

   Modification: The following subsection has been added to the end of Section 1 (marked in red):

Related Work

In addition to conventional statistical and deep learning methods for time series anomaly detection, recent studies have explored advanced neural architectures tailored to power system monitoring. For example, residual-based attention Physics-informed Neural Networks (PINNs) [21] have been proposed for spatiotemporal aging assessment of transformers in renewable energy environments, incorporating prior physical knowledge into the network structure. Other works have focused on adversarial robustness in smart grids, including the EVADE framework [22] for targeted adversarial false data injection (FDI), LESSON [23] for multi-label FDI detection, and joint adversarial-FDI models [24] for enhanced state estimation resilience.

While these studies provide valuable advances in security-aware and physics-driven modeling, they typically target classification or estimation under adversarial perturbations, and often rely on explicit physical constraints or label supervision. In contrast, our work addresses early-stage fault detection under nonstationary SCADA conditions through a residual-aware spatiotemporal graph learning framework. The proposed method emphasizes dynamic topology learning, interpretable residual-based indicators, and scalable fault diagnosis without requiring detailed physical models or attack assumptions.

5. **Comment 5:** *"Although experimental results are provided, more in depth comparison and analysis should be given in the manuscript."*

   Response: We thank the reviewer for this valuable suggestion. In the revised manuscript, the experimental results section (Section 5) has been expanded with additional comparative analysis and interpretation. Specifically, we have:

   (1) included deeper discussion on the convergence behavior and prediction stability across models based on RMSE curves (Fig. 2); (2) added a paragraph analyzing model robustness under noisy versus clean test intervals (Fig. 3); (3) provided observations about failure cases and subsystem-specific detection performance in the anomaly detection task; (4) summarized key takeaways from the comparison results to help readers understand the practical advantages of our STGNN framework.

   These enhancements aim to provide a more comprehensive view of the model's strengths and behavior under different operational conditions.

   Modification:

   In Section 5.2 ("Model Performance Evaluation"), we added:

   Table 2 reports the prediction results in terms of Root Mean Square Error (RMSE), Mean Absolute Percentage Error (MAPE), and maximum node-level residuals. The proposed STGNN outperforms all baselines across all metrics. Compared to LSTM and GCN-LSTM, STGNN achieves substantial improvements in both absolute error reduction and relative percentage accuracy. This indicates the advantage of jointly modeling dynamic graph structures and residual-enhanced temporal dependencies, especially for multivariate SCADA signals with nonstationary behaviors. In particular, the lowest Max Error value reflects the model's stability in forecasting high-impact signals such as winding temperature and partial discharge, which are critical for early-stage degradation detection.

Figure 2 shows the RMSE convergence curves across 50 training epochs. STGNN achieves the lowest plateau RMSE and converges faster than the baselines, benefiting from the spatiotemporal coupling of graph attention and residual-enhanced temporal encoding. Notably, the convergence curve of STGNN is smoother and more stable than those of GCN-LSTM and StemGNN, which exhibit larger oscillations throughout training. This suggests that the residual-enhanced GRU module effectively stabilizes gradient updates during backpropagation, thereby accelerating convergence and reducing the risk of overfitting or premature plateaus. In addition, the dynamic graph attention mechanism allows the model to flexibly adapt to evolving inter-sensor dependencies at each time step, which contributes to better generalization and predictive consistency. From a practical standpoint, fast and stable convergence is particularly beneficial in online or incremental training scenarios, where the model must rapidly adapt to new SCADA data without extended retraining. These results demonstrate that the architectural design of STGNN not only improves final forecasting accuracy, but also enhances optimization behavior during the training process.

In Section 5.3 ("Health Indicator and Anomaly Detection Analysis"), we added:

Figure 4 presents a representative HI sequence from the test set, comparing the outputs of different models against the annotated ground truth fault interval. The STGNN model exhibits a distinct upward deviation in HI before the onset of failure, enabling early fault warning. This sharp rise reflects the model's sensitivity to subtle degradation patterns, which accumulate gradually before becoming critical. By contrast, LSTM and GCN-LSTM show flatter or delayed changes in their HI curves, indicating reduced responsiveness to weak early-stage deviations.

Moreover, the temporal alignment of HI spikes from STGNN closely matches the onset of true failure intervals, enabling better fault localization in time. This precise timing is essential for real-world SCADA systems where maintenance scheduling or alert escalation must be both early and trustworthy.

To quantify detection performance, we compute standard classification metrics: Accuracy, Precision, Recall, and F1-score, over the entire test set. As reported in Fig. 3, STGNN achieves F1-scores of 0.91 and 0.82 under clean and noisy conditions respectively, outperforming all baselines. Importantly, the recall metric of STGNN remains high even under noise injection, while other models (especially LSTM) suffer significant drops. This suggests that the residual-based HI—when paired with dynamic attention graphs—is more robust to noise, as it emphasizes structural deviations rather than isolated signal fluctuations.

To evaluate the stability of the HI thresholding scheme, we conduct a sensitivity analysis across multiple quantile settings ranging from 90% to 99%. Results indicate that the 95th percentile provides the optimal trade-off between false positives and missed detections. We observe that STGNN maintains consistent F1-scores across different thresholds, while LSTM and GCN-LSTM exhibit greater performance fluctuations. This reflects the stability of the residual signal distribution generated by our model, making it more suitable for integration into alarm-based monitoring workflows.

In real-world deployments, robustness to noise, stable decision thresholds, and early fault

sensitivity are essential features. The results in this section demonstrate that the proposed STGNN design satisfies these requirements through its combination of temporal residual modeling and adaptive graph attention.

6. **Comment 6:** *"What are the possible shortcomings of the research in this paper if any? Add a section on the limitations of the work and future work in this paper."*

   Response: We thank the reviewer for pointing out the importance of acknowledging the limitations of our work. A dedicated subsection titled *"Limitations and Future Work"* has been added at the end of Section 6. This new section discusses three key aspects: (i) the current reliance on synthetic SCADA data and the need for real-world validation; (ii) the assumption of full variable observability at all time steps; and (iii) the lack of interpretability tools for diagnosing graph-level decision behavior. These points also serve as the basis for outlining future directions for model deployment, semi-supervised learning, and explainability enhancement.

   Modification:

   A new subsection has been added at the end of Section 6 to address limitations and future directions.

Limitations and Future Work

While the proposed STGNN framework demonstrates strong performance in forecasting and anomaly detection, several limitations remain to be addressed in future work.

- **Synthetic Dataset Generalizability.** All experiments were conducted using a synthetic SCADA dataset designed to emulate realistic transformer behavior. Although care was taken to simulate various fault types and subsystem dynamics, real-world variability may involve unmodeled patterns, non-Gaussian noise, and external disturbances. Validation on real SCADA logs from industrial substations will be pursued in future studies to assess deployment feasibility.
- **Full Observability Assumption.** The current model assumes that all sensor variables are fully observed at each time step. However, in real-world SCADA environments, signal dropout, packet loss, and asynchronous sampling often occur. Future work will explore spatiotemporal imputation strategies and semi-supervised graph learning methods to address missing or sparse observations.
- **Model Interpretability.** Although the proposed residual-based health indicator improves interpretability over pure black-box forecasting models, the internal graph attention mechanisms remain opaque. Further efforts will be made to incorporate graph explainability tools (e.g., GNNExplainer or counterfactual attention tracing) to interpret node-level contributions to fault detection decisions.

These limitations motivate future work toward real-world validation, robustness to incomplete data, and greater transparency in decision-making for power system diagnostics.

7. **Comment 7:** *"The manuscript contains a number of linguistic errors that hinder comprehension. The authors are advised to make careful revisions and improvements."*

   Response: We thank the reviewer for highlighting the importance of clear and precise language. A thorough proofreading of the entire manuscript has been conducted to correct typographical issues, improve grammatical accuracy, and enhance the overall fluency of the text. Domain-specific expressions and technical terms have also been refined to ensure clarity for a broad readership. In addition, all revised content has been reviewed by a native English-speaking academic with expertise in power systems and machine learning to ensure linguistic correctness.

   Modification: Throughout the manuscript, grammatical and stylistic improvements have been made. These include:

   - Refining technical phrases for clarity (e.g., "attention-based adjacency matrix" instead of "attention relation graph").
   - Correcting tense and voice usage in methodology descriptions (e.g., passive voice for model steps).
   - Eliminating redundant or ambiguous terms in the Introduction and Experimental sections.
   - Improving figure and table captions to provide clearer guidance to the reader.

The authors would like to extend our heartfelt gratitude to the reviewer for their thorough and insightful comments, which have been instrumental in enhancing the quality and clarity of our manuscript. We deeply value the time and effort they have dedicated to providing constructive feedback and thoughtful suggestions, which have helped us to refine the content and presentation of our work further.

Response to Reviewer No. 4:

Ms. No. PONE-D-25-34707

"Residual-Aware Health Prediction of Power Transformers via Spatiotemporal Graph Neural Networks"

We sincerely appreciate the reviewers' time and constructive feedback. In response to the comments from Reviewer 4, we have revised the manuscript to improve both its clarity and technical accuracy. For ease of review, all modifications addressing Reviewer 4's suggestions are highlighted in red throughout the revised manuscript. We are grateful for the insightful remarks, which have led to substantial improvements in the quality and presentation of our work.

1. **Comment:** *"Currently, the experimental validation relies solely on synthetic datasets, which lack real-world data verification from actual industrial environments..."*

   Response: We appreciate the reviewer's insightful observation regarding the importance of real-world validation. In this work, a synthetic SCADA dataset was developed to simulate key operational patterns and fault modes across five transformer subsystems. This dataset incorporates dynamic behaviors and multi-type anomalies commonly observed in industrial environments, and was designed in consultation with transformer condition monitoring standards such as IEC 60076-7. Nevertheless, we acknowledge that synthetic data cannot fully represent the noise characteristics and unpredictability of real-world scenarios. To address this, we have explicitly discussed the limitations of synthetic evaluation in a newly added section titled *Limitations and Future Work*. In this section, we clarify our ongoing efforts to access industrial SCADA logs via institutional partnerships, and emphasize our plan to test the proposed STGNN framework on real historical and streaming data in future studies.

   Modification: While the synthetic SCADA dataset used in this study provides a controlled environment for model validation, it may not capture the full variability and noise patterns of real industrial transformer systems. Future work will focus on extending the evaluation to historical and online SCADA data collected from operational substations. Collaborations are currently being established with grid operators to obtain field data, which will allow the STGNN framework to be benchmarked under authentic conditions. Additionally, methods for domain adaptation and transfer learning will be explored to enhance generalization across different transformer types and deployment environments. A new subsection titled **6.1 Limitations and Future Work** has been added to the Conclusion section. It reads as follows:

   - **Synthetic Dataset Generalizability.** All experiments were conducted using a synthetic SCADA dataset designed to emulate realistic transformer behavior. Although care was taken to simulate various fault types and subsystem dynamics, real-world variability may involve unmodeled patterns, non-Gaussian noise, and external disturbances. Validation on real SCADA logs from industrial substations will be pursued in future studies to assess deployment feasibility.
   - **Full Observability Assumption.** The current model assumes that all sensor variables are fully observed at each time step. However, in real-world SCADA environments, signal dropout, packet loss, and asynchronous sampling often occur. Future

work will explore spatiotemporal imputation strategies and semi-supervised graph learning methods to address missing or sparse observations.

- **Model Interpretability.** Although the proposed residual-based health indicator improves interpretability over pure black-box forecasting models, the internal graph attention mechanisms remain opaque. Further efforts will be made to incorporate graph explainability tools (e.g., GNNExplainer or counterfactual attention tracing) to interpret node-level contributions to fault detection decisions.

2. **Comment:** *"The manuscript only compares the proposed method with LSTM and GCN-LSTM... more diverse comparison experiments..."*

   Response: We thank the reviewer for this important suggestion. In the revised manuscript, we have extended our baseline comparison by including two additional advanced models—**StemGNN** and **TempGNN**—which are representative spatiotemporal graph neural networks commonly used in recent literature. These models were selected for their relevance to multivariate time-series forecasting tasks in sensor networks and infrastructure monitoring.

   Quantitative results of these models have been added to Table 2 and their convergence behaviors are illustrated in Figure 2. Further discussion on their comparative performance is provided in Section 5.2. Our findings demonstrate that the proposed STGNN consistently outperforms all four baselines in terms of both prediction accuracy and anomaly detection robustness.

   Regarding classical statistical methods such as PCA or SVM, we note that these techniques are generally not well-suited for high-dimensional temporal forecasting tasks, especially where multivariate interactions and temporal dependencies play a critical role. Nevertheless, we have added a clarifying footnote to Table 2 explaining this distinction and noting the potential for future benchmarking under classification settings.

   Modification:

   Table 2 reports the prediction results in terms of Root Mean Square Error (RMSE), Mean Absolute Percentage Error (MAPE), and maximum node-level residuals. The proposed STGNN outperforms all baselines across all metrics. Compared to LSTM and GCN-LSTM, STGNN achieves substantial improvements in both absolute error reduction and relative percentage accuracy. This indicates the advantage of jointly modeling dynamic graph structures and residual-enhanced temporal dependencies, especially for multivariate SCADA signals with nonstationary behaviors. In particular, the lowest Max Error value reflects the model's stability in forecasting high-impact signals such as winding temperature and partial discharge, which are critical for early-stage degradation detection.

   Notably, the convergence curve of STGNN is smoother and more stable than those of GCN-LSTM and StemGNN, which exhibit larger oscillations throughout training. This suggests that the residual-enhanced GRU module effectively stabilizes gradient updates during backpropagation, thereby accelerating convergence and reducing the risk of overfitting or premature plateaus. In addition, the dynamic graph attention mechanism allows the model to flexibly adapt to evolving inter-sensor dependencies at each time step, which contributes to better generalization and predictive consistency.

From a practical standpoint, fast and stable convergence is particularly beneficial in online or incremental training scenarios, where the model must rapidly adapt to new SCADA data without extended retraining. These results demonstrate that the architectural design of STGNN not only improves final forecasting accuracy, but also enhances optimization behavior during the training process.

3. **Comment:** *"The quality of Figure 1 in the manuscript is suboptimal... A clear, professional figure is crucial..."*

   Response: Thank you for pointing this out. We have carefully redesigned Figure 1 to ensure improved clarity, academic presentation quality, and readability. The updated figure includes higher resolution, optimized font sizes, thicker line weights, and better color contrast. Annotations have also been revised to be more concise and professional.

   Modification: Figure 1 has been replaced with a redesigned version following the reviewer's suggestions.

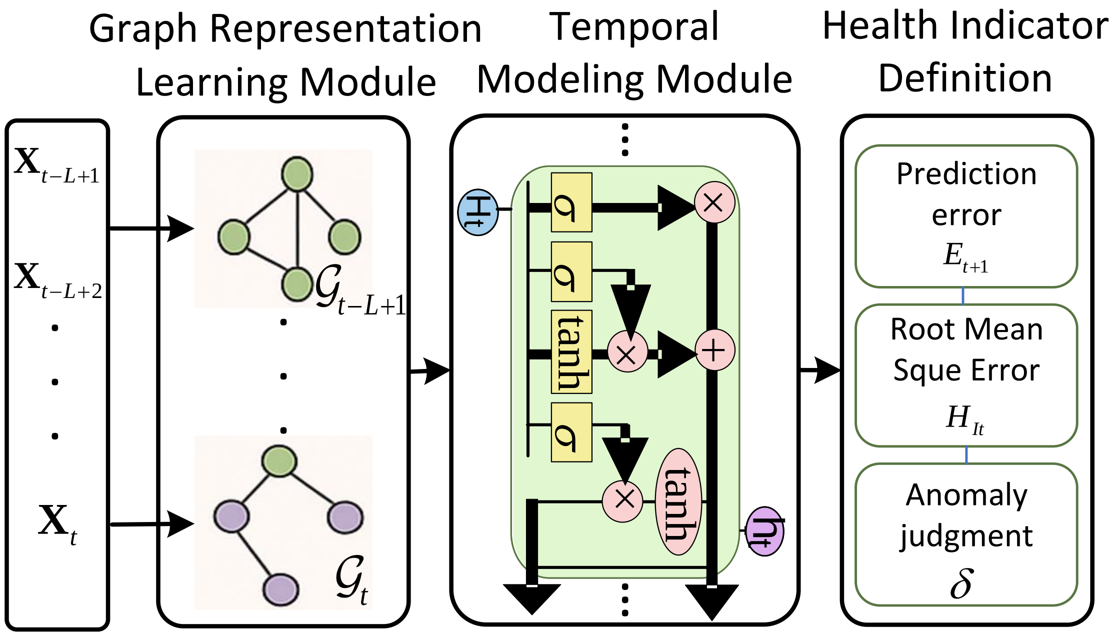

Figure 1: Overall model architecture combining graph representation learning, temporal modeling via GRU, and health indicator computation for anomaly detection.

4. **Comment:** *"Some technical details on how spatiotemporal dependencies and residual connections are modeled are not sufficiently explained..."*

   Response: We thank the reviewer for this valuable suggestion. In the revised manuscript, we have significantly enhanced the explanation of how spatial and temporal dependencies are captured in our STGNN framework. Specifically:

   - We clarified that the spatial dependencies are learned from dynamically constructed graphs using a soft attention mechanism, where each time-varying adjacency matrix $\mathbf{A}_t$ combines static topology, feature similarity, and adaptive attention scores.

- We provided more detailed mathematical formulations for the graph convolution operations, explicitly using the Chebyshev spectral approximation to implement localized filtering over dynamic graphs.
- We added further explanation on the role of residual-enhanced GRUs in temporal modeling. The residual connection improves gradient stability and facilitates the capture of small but persistent deviations in SCADA sequences, which are often early indicators of faults.

These additions can be found in the updated Section 3.1,Section 3.2 and Section 3.3, with supporting equations and architecture diagrams. We hope this significantly improves the clarity and interpretability of the proposed method.

Modification: To address the reviewer's concern, we have significantly expanded Section 3.1 (Model Architecture Design), Section 3.2 (Graph Representation Learning Module), and Section 3.3 (Temporal Modeling Module) to enhance the clarity and technical depth of the spatiotemporal modeling framework.

- In Section 3.1, a detailed overview of the full architecture is now provided, including explicit descriptions of each module's role and their interconnections. We also included the design motivations behind the adoption of dynamic attention-based graphs, Chebyshev spectral convolution, and residual-enhanced temporal encoding.
- In Section 3.2, we have added formal equations for the attention-based dynamic graph construction (Eq. 15–16) and the Chebyshev spectral graph convolution (Eq. 17), along with intuitive justifications of their selection for transformer monitoring scenarios.
- In Section 3.3, the temporal modeling process is explained in full, including GRU state updates (Eq. 19), residual-based forecasting (Eq. 20–21), and the node-aware weighted loss function (Eq. 22). We also highlight how residual connections improve sensitivity to long-tail degradation patterns.

Each equation and figure has now been properly numbered and referenced in the text to improve readability and reproducibility. These updates aim to provide a transparent and rigorous account of the proposed method's internal mechanisms.

Once again, we are very grateful to Reviewer 4 for their encouraging comments and insightful suggestions. We believe the manuscript has been significantly improved based on your feedback, and we hope the revised version now meets the journal's standards.
